# Supplementary material for: A Molecular Survey on Neglected Gurltia paralysans and Aelurostrongylus abstrusus Infections in Domestic Cats (Felis catus) from Southern Chile
Source: Pathogens. 2021 Sep 15;10(9):1195. doi: 10.3390/pathogens10091195 (PMC8468845; doi:10.3390/pathogens10091195)
Supplement: Supplementary file 1 [file pathogens-10-01195-s001.zip › pathogens-1363818-supplementary.pdf]

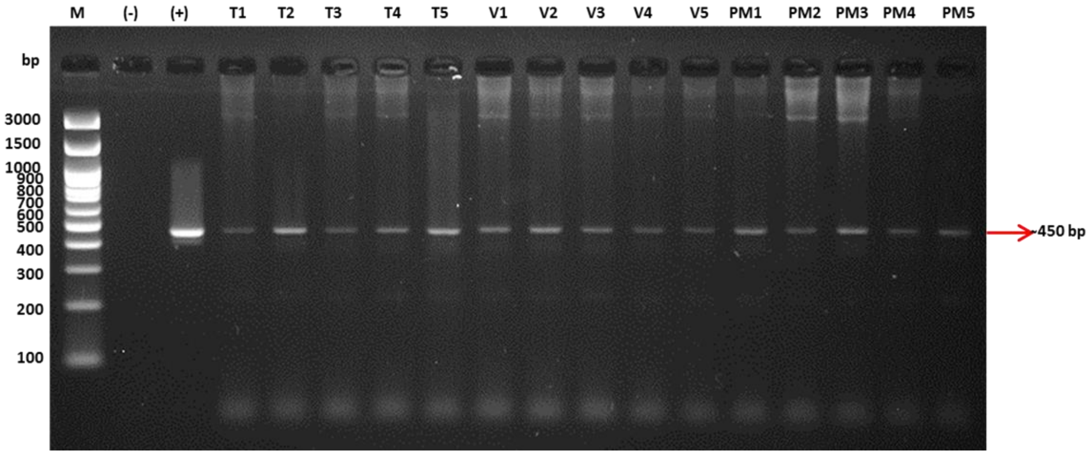

Figure S1. Amplification of common DNA sequence of the Metastrongyloidea superfamily, obtained in a PCR examination with blood DNA samples of domestic cats from the cities of Temuco, Valdivia and Puerto Montt, Southern Chile. M: Molecular Weight Marker. T1 → T5: Samples from the city of Temuco. V1 → V5: Samples from the city of Valdivia. PM1 → PM5: Samples from the city of Puerto Montt. (-): Negative control. (+): Positive control. bp: base pairs. Molecular target (red arrow): approximately 450 base pairs (bp).

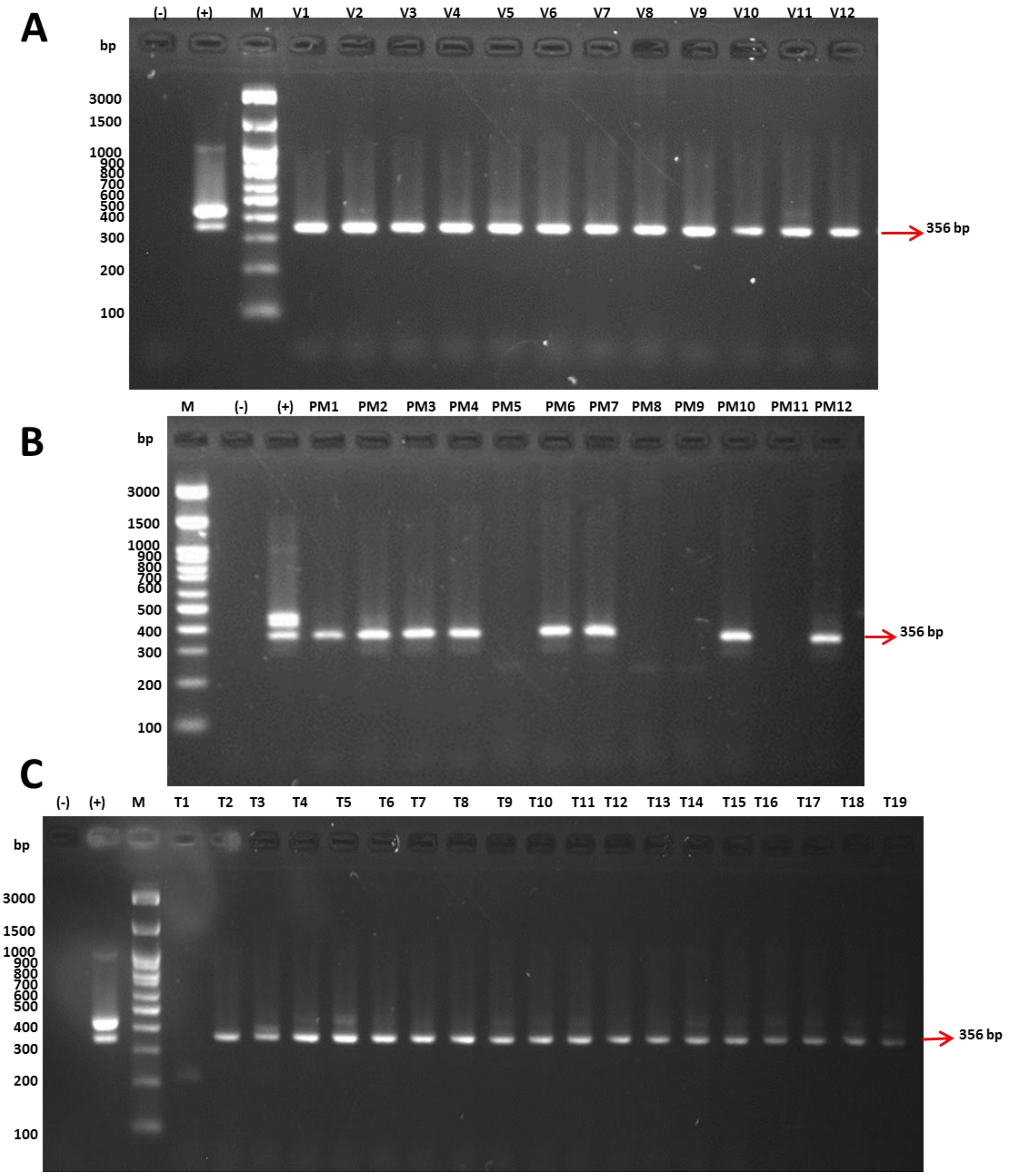

Figure S2. Molecular detection of *Gurltia paralysans* by semi-nested PCR in domestic feline blood DNA samples. M: Molecular Weight Marker. A: DNA samples of domestic cat blood from the City of Valdivia, Los Ríos Region, Chile (V1 → V12). B: DNA samples from domestic cat blood from the city of Puerto Montt, Los Lagos Region, Chile (PM1 → PM12). C: DNA samples from domestic cat blood from the city of Temuco, La Araucanía Region, Chile (T1 → 19). (-): Negative control. (+): Positive control. bp: base pairs. Molecular target (red arrow): 356 bp.

Table S1. Genetic sequence analysis of 7 samples for *Gurltia paralysans* and 2 samples for *Aelurostrongylus abstrusus* from domestic felines from southern Chile.

Sample	Description	Max. Score	Total Score	Query Coverage	E-value	% Identity	Accession
PT37	*Gurltia paralysans* 18S ribosomal RNA gene, partial sequence; internal transcribed spacer 1, 5.8S ribosomal RNA gene, and internal transcribed spacer 2 complete sequence; and 28S ribosomal RNA gene, partial sequence.	459	459	100%	$3x10^{-125}$	100%	JX975484.2
PT46	*Gurltia paralysans* 18S ribosomal RNA gene, partial sequence; internal transcribed spacer 1, 5.8S ribosomal RNA gene, and internal transcribed spacer 2 complete sequence; and 28S ribosomal RNA gene, partial sequence.	460	460	100%	$1x10^{-125}$	100%	JX975484.2
PT52	*Gurltia paralysans* 18S ribosomal RNA gene, partial sequence; internal transcribed spacer 1, 5.8S ribosomal RNA gene, and internal transcribed spacer 2 complete sequence; and 28S ribosomal RNA gene, partial sequence.	640	640	99%	$2x10^{-179}$	100%	JX975484.2
T22	*Gurltia paralysans* 18S ribosomal RNA gene, partial sequence; internal transcribed spacer 1, 5.8S ribosomal RNA gene, and internal	558	558	100%	$4x10^{-155}$	99.7%	JX975484.2

	transcribed spacer 2 complete sequence; and 28S ribosomal RNA gene, partial sequence.						
T38	*Gurltia paralysans* 18S ribosomal RNA gene, partial sequence; internal transcribed spacer 1, 5.8S ribosomal RNA gene, and internal transcribed spacer 2 complete sequence; and 28S ribosomal RNA gene, partial sequence.	617	617	100%	8×10^{-173}	100%	JX975484.2
T58	*Gurltia paralysans* 18S ribosomal RNA gene, partial sequence; internal transcribed spacer 1, 5.8S ribosomal RNA gene, and internal transcribed spacer 2 complete sequence; and 28S ribosomal RNA gene, partial sequence.	614	614	100%	1×10^{-171}	100%	JX975484.2
PT59	*Gurltia paralysans* 18S ribosomal RNA gene, partial sequence; internal transcribed spacer 1, 5.8S ribosomal RNA gene, and internal transcribed spacer 2 complete sequence; and 28S ribosomal RNA gene, partial sequence.	623	623	100%	2×10^{-174}	100%	JX975484.2
T22_A	*Aelurostrongylus abstrusus* 28S rRNA gene	263	263	100%	2×10^{-66}	89.40%	AM039759.1

T38_A	*Aelurostrongylus abstrusus* 28S rRNA gene	316	316	100%	$2x10^{-82}$	90.36%	AM039759.1
